# Supplementary material for: Natural Bioactives: Back to the Future in the Fight against Human Papillomavirus? A Narrative Review
Source: J Clin Med. 2022 Mar 7;11(5):1465. doi: 10.3390/jcm11051465 (PMC8911515; doi:10.3390/jcm11051465)
Supplement: Supplementary file 1 [file jcm-11-01465-s001.zip › jcm-1583072-supplementary.pdf]

Table S1. Summary of plant-derived formulations that have been utilized in clinical trials against HPV, listed in chronological order, by chemical group association.

	Text Reference Number/Composition	Administration	Type of Study	Outcome	Bibliography Ref./Year/Country
Indole-3-carbinol-based formulations	**No.1**<br>**Indole-3-carbinol**<br>**(**I-3-C; Theranaturals, Inc., Orem, Utah)	• Adults: 200 mg I-3-C / orally/ twice daily<br>• Children: 10 mg I-3-C / kg / orally / twice daily	Phase I trial<br>(N= 18 patients with recurrent respiratory papillomatosis)	On average follow-up of 14.6 months:<br>• Cessation of papilloma growth and no surgery in 33 % (6 out of 18) on I3C<br>• reduced papilloma growth rate in 33 % (6 out of 18) on I3C<br>• no clinical response to I3C in 33 % (6 out of 18) on I3C<br>• Correlation of ratio of hydroxylation of estradiol by I3C and clinical response.<br>• Safe and well tolerated also in children.	[123]<br>1998<br>USA
	**No.2**<br>**Indole-3-carbinol (I-3-C)**	• Group 1 (n = 9): I-3-C 200 mg / orally / daily/ 12 weeks<br>• Group 2 (n= 8): I-3-C 400 mg / orally / daily/ 12 weeks<br>• Group 3 (n = 10): placebo / orally / daily/ 12 weeks	Placebo-Controlled Trial<br>(N= 27 patients with histologically confirmed CIN II – CIN III)	• Statistically significant complete regression in 4 out of 8 patients on I-3-C 200 mg/day and in 4 out of 9 patients on I-3-C 400 mg/day, compared with placebo.<br>• HPV clearance in 7 of 8 on 200 mg/day, and in 8 out of 9 on 400 mg/day (in 7 out of 10 on placebo)<br>• Change of the 2/16a-hydroxyestrone ratio.	[124]<br>2000<br>USA
	**No.3**<br>**Indole-3-carbinol**<br>**(**I-3-C; supplied by Theranaturals, Inc., Orem, Utah)	• Adults: 200 mg I-3-C / orally/ twice daily<br>• Children: 10 mg I-3-C / kg / orally / twice daily	Prospective, open-label clinical trial<br>(N= 33 patients with recurrent papillomatosis)	On average follow-up of 4.8 years:<br>• Remission of papillomatous growth and no surgery in 11 (33%) patients on I3C.<br>• Reduction in papillomatous growth and less frequent surgery in 10 (30%) patients on I3C.	[125]<br>2004<br>USA

			• No clinical response in 12 (36%) patients on I3C.	
**No.4**<br>**Diindolylmethane (DIM) dietary supplement**<br>**(**DIM®, stable form of Indole-3-carbinol, supplied by BioResponse, Boulder, CO)	• Group 1 (n = 45): DIM 2 mg/kg/day / orally / 12 weeks<br>• Group 2 (n = 19): placebo / orally / daily / 12 weeks	Randomized double blind placebo controlled phase III trial<br>(N= 64 patients with CIN II or CIN III)	No statistical difference between treatment and placebo<br>• CIN decreased by 1–2 grades or to normal in 47% (21 subjects) on DIM<br>• Improved Pap smear in 49% (22/45) on DIM<br>• Improved colposcopy in 56% (25 subjects) on DIM.<br>• Complete colposcopic response in 9% (4 subjects) on DIM.<br>• Stratification by level of dysplasia, age, race, HPV status, tobacco use, contraceptive used did not alter the results.<br>No systemic toxicities	[126]<br>2010<br>USA
**No.5**<br>**Diindolylmethane (DIM) dietary supplement**<br>**(**DIM®, stable form of Indole-3-carbinol, supplied by BioResponse, Boulder, CO)	• Group 1 (n = 373): DIM 150 mg / orally / daily/ 180 days<br>• Group 2 (n = 178): placebo / orally / daily/ 180 days	Double-blind, randomised, controlled, primary prevention trial<br>(N= 551 patients with low-grade cytological abnormalities)	DIM oral supplementation had no statistically relevant effect on cytology or infection compared to placebo:<br>• Development of CIN2 or worse in 9% on DIM and 12% on placebo (risk ratio (RR) 0.7).<br>• Development of CIN3 or worse (RR 0.9) in 4.6% on DIM and 5.1% on placebo<br>• No sign of disease in 27.3% on DIM and 34.3% on placebo at 6 months (RR 0.8).<br>Supplementation was well tolerated	[127]<br>2012<br>UK
**No.6**<br>**Diindolylmethane (DIM) intravaginal suppository**	• Group 1 (n = 26): DIM 200 mg (2 suppositories) / intravaginally / daily/ 180 days	Double-blind randomized placebo controlled multicenter clinical trial phase IIa<br>(N= 78 patients with CIN I-II)	• Statistically relevant CIN regression in 90,5 % (19 out of 21) and 100% (19 out of 19) of patients after 90–180 days of treatment with 100 and 200 mg/day, respectively.	[128]<br>2015<br>RUSSIA

		• Group 2 (n = 26): DIM 100 mg (1 suppository) / intravaginally / daily/ 180 days<br>• Group 3 (n = 26) placebo (one suppository) / intravaginally / daily/ 180 days		• Moderate AEs registered in 40.0 % of patients (10 of 25 subjects, CI 95 %:21.1–61.3 %) on DIM 100 mg/day<br>• Moderate AEs registered in 42.0 % of patients (10 of 24 subjects, CI 95 %: 22.1–63.4 %) on DIM 200 mg/day	
Curcumin-based formulations	**No.1 Curcumin** (99,3 % pure diferuloylmethane)	• Group 1: curcumin 500 mg / orally / day / 3 months<br>• Group 2: curcumin 1000 mg / orally / day / 3 months<br>• Group 3: curcumin 2000 mg / orally / day / 3 months<br>• Group 4: curcumin 4000 mg / orally / day / 3 months<br>• Group 5: curcumin 8000 mg / orally / day / 3 months	Phase I Clinical trial (N= 4 patients with CIN)	• CIN normalization in 1 out of 4 cases on Curcumin 500 mg<br>• No toxicity up to 8,000 mg/day/3 months	[103] 2001 CHINA
	**No.2 Basant™ polyherbal cream**:<br>• Curcumin (95% pure diferuloyl methane (EE)-1,7-bis(4-hydroxy-3-methoxyphenyl)-	• Group 1: Basant™ cream (5 ml) / intavaginal / day / 30 days<br>• Group 2: placebo cream) / intravaginal / day / 30 days	Placebo-controlled Phase II Randomized Study (N= 280 HPV-positive women without high grade cervical neoplasia)	Basant™:<br>• HPV clearance in 87.7% of patients (not statistically significant compared to placebo) in early stages LSIL. Statistically significant when HPV clearance was compared to the two placebo arms (cream and capsule; N=128)	[155] 2013 INDIA

1,6-heptadiene-3,5-dione)<br>• purified extracts of *Emblica officinalis* and of *Aloe barbadensis*<br>• purified saponins from *Sapindus mukorossi*<br>• Rose water<br>**Curcumin soft gelatine capsules**	• Group 3: 500 mg curcumin (1 gelatine capsule) / intravaginal / day / 30 days<br>• Group 4: placebo gelatine capsule / intravaginal / day / 30 day		• 38 days median duration of HPV clearance<br>• AEs: Mild to moderate vaginal irritation.<br>Curcumin:<br>• HPV clearance (81.3%, not statistically significant compared to placebo).<br>• 39 day median duration of HPV clearance	
**No.3**<br>**Basant™ intravaginal capsules**:<br>• Curcumin (95% pure diferuloyl methane (E,E)-1,7-bis(4-hydroxy-3-methoxyphenyl)-1,6-heptadiene-3,5-dione)<br>• purified extracts of *Emblica officinalis*, *Azadirachta indica* leaves, *Aloe barbadensis*<br>• Excipients: citric acid, sorbitol, microcrystalline cellulose, sodium starchglycolate, starlac,	• Group 1: 2 capsules (250 mg each) / intravaginally / day / 30 days<br>• Group 2: Placebo / intravaginally / day / 30 days	Placebo-controlled Phase I Clinical Trial<br>(N=11 HPV 16-positive women, without high grade cervical neoplasia)	• HPV clearance in 100% on Basant™<br>• Normal or improved Pap smear in 1 out of 11 and 10 out of 11 on Basant™ and Placebo, respectively	[156]<br>2015<br>INDIA

	crospovidones, sodium alginate				
Catechin-based formulations	**No.1**<br>**Polyphenon E (poly E)**<br>• decaffeinated and enriched green tea catechin extract, containing 85–95% total catechins, with 56–72% as EGCG<br>• oral capsules containing 200 mg EGCG / capsule<br>• Provided by green tea producing Mitsui Norin Co. Ltd.	• Group 1 (n= 27): poly E ointment (200 mg EGCG) / daily / intravaginally / 8-12 weeks<br>• Group 2 (n= 8): poly E ointment + EGCG capsule (200 mg EGCG) / daily / orally - intravaginally / 8-12 weeks<br>• Group 3 (n= 6): poly E capsule (200 mg EGCG) / daily / orally / 8-12 weeks<br>• Group 4 (n= 10): EGCG capsule (200 mg EGCG) / daily / orally / 8-12 weeks<br>• Group 5 (n= 39): Placebo	Phase I clinical trial (N= 90 patients with Chronic cervicitis or Mild or Moderate or Severe dysplasia)	• Statistically significant disappearance / decrease of lesion in 74% on Poly E ointment compared to placebo<br>• Statistically significant disappearance / decrease of lesion in 75% on Poly E ointment + EGCG capsule<br>• Statistically significant disappearance / decrease of lesion in 50% on Poly E capsule<br>• Statistically significant disappearance / decrease of lesion in 60% on EGCG capsule<br>• Mild inflammatory responses and abnormalities<br>• No systemic toxicity	[145]<br>2003<br>SOUTH COREA
	**No.2**<br>**Polyphenon E (poly E)**<br>• decaffeinated and enriched green tea catechin extract, containing 85–95% total catechins,	• Group 1 (n= 50): poly E (800 mg of EGCG) / daily / oral / 4 months	Phase II Randomized, Double-blind, Placebo Controlled Trial (N= 98 patients with persistent high risk HPV Infection and CIN I)	• No statistically relevant promotion of neither clearance of persistent high-risk HPV or normal hystopathology.<br>• Persistent oncogenic HPV with histopathologic evidence of progression in 6	[146]<br>2014<br>USA

	with 56–72% as EGCG<br>• oral capsules containing 200 mg EGCG / capsule provided by the green tea producing Mitsui Norin Co. Ltd.	• Group 2 (n= 48): placebo / daily / oral / 4 months		(14.6%) and 3 (7.7%) in the Polyphenon E and placebo arms, respectively.<br>• Poly E was acceptable, safe and well tolerated, but AEs more frequent on Poly E	
Other Polyherbal-based formulations	**No.1**<br>**Praneem polyherbal (**500 mg):<br>• purified leaf extract of *Azadirachta indica*, 80 mg;<br>• purified saponins from *Sapindus muckerossi*, 40 mg;<br>• *Mentha citrate* oil, 20 mg;<br>• Quinine Hydrochloride 30 mg;<br>• Excipients: citric acid, sorbitol, microcrystalline cellulose, sodium starchglycolate, starlac, crospovidones, sodium alginate	• Group 1 (n= 10): Praneem 500 mg (1 tablet) / daily / intra-vaginally / 30-60 days<br>• Group 2 (n= 10): Placebo	Open-label, placebo-controlled clinical trial (N= 20 HPV 16 positive patiens, with or without LSIL)	• Clearance of HPV 16 in 60% on Praneem for 30 days<br>• Clearance of HPV 16 in 20% on Praneem for 60 days<br>• Overall clearance rate (80%) statistically significant compared to 10% spontaneous clearance rate on placebo<br>• .Marked improvement of clinical symptoms and cytological abnormalities in cleared patients.	[157]<br>2009<br>INDIA

Table S2. Summary of phytochemicals that have been screened against HPV listed in alphabetical order within the different chemical groups (↑: upregulation/induction/increase; ↓: downregulation/reduction/decrease).

Compound	Dietary Sources	Model		IC50	Mechanism of Action	References
Apigenin (Polyphenols, Flavonoids, Flavones)	Onion, parsley, oranges, tea, wheat sprouts, chamomile, Chinese cabbage, bell pepper, garlic, bilimbi fruit, guava, wolfberry leaves	In vitro	CaSki, HeLa, C33A	10 – 76 – 40 μM	↑ G1 phase growth arrest, p53, Apoptosis, p21/WAF1, Fas/APO-1 and caspase-3.<br>↓ Proliferation, traslocation, Bcl-2<br>Modification of cell motility (nterference with gap junctions)	[55–57]
Baicalein (Polyphenols, Flavonoids, Flavones)	Dry root of *Scutellaria baicalensis*	In vitro	HeLa, SiHa, C33A	-	↓ Colony-forming capacity, Invasion, p21/p27, Hedgehog/Gli signaling pathway, NF-κB pathway, TGFβ pathway, Proliferation, mTOR/p70S6K signal pathway, AKT/mTOR signal pathway, Notch-1/Hes-1 (Hes-5), Cell migration, miR-19a-3p<br>↑ CyclinD1, Cell cycle arrest at G0/G1, Apoptosis, Bax/Bcl-2 ratio, Fas, FasL, Caspase-8, E-cadherin	[195–198]
		In vivo	Nude mice (with cervical cancer xenograft)	-	↓ Long non-coding RNA	
			Kun Ming mice with mouse U14 cervical cancer cell line	-	↓ Tumor weight<br>↑ Thymus weight and spleen weight, Bax/Bcl-2 ratio	
Butein (Polyphenols, Chalcones)	*Toxicodendron vernicifluum, Semecarpus anacardium, Dalbergia odorifera, Caragana jubata,* and *Rhusverniciflua* sp.	In vitro	HeLa, MCF-7, ME-180, SiHa, C33A	-	Possible gene regulatory role	[199–201]
		In vivo	HeLa xenograft mouse	-		
Caffeic Acid (Polyphenols, Hydroxycinnamic acids)	Coffee, fruits, vegetables, and olive oil	In vitro	HeLa, ME-180	-	↓ Mitochondrial membrane potential<br>↑ S and G2/M growth arrest, lipid peroxidative markers (thio-barbituric acid reactive substances, conjugated dienes, and lipid hydroperoxide), ROS, apoptotic morphological changes and cancer cell death by binding to HDAC	[35,36]
Calycosin (Polyphenols, Flavonoids, Isoflavones)	Radix astragali	In vitro	HeLa, CaSki, SiHa, C33A	-	↓ Invasion, miR-375, Cell viability, Apoptosis	[202]
Chrysin (Polyphenols, Flavonoids, Flavones)	*Scutellaria discolor*, passion flower, *Passiflora* spp., propolis, honey, mushroom, honeycomb	In vitro	HeLa	14.2 μM	↓ Akt signaling, Proliferation<br>↑ Apoptosis, Caspases, p38, NFkB/p65, Cell cycle arrest	[203,204]

Compound	Dietary Sources	Model		IC50	Mechanism of Action	References
Curcumin and MS17 curcumin analogue 1,5-Bis(2-hydroxyphenyl)-1,4-pentadiene-3-one	Rhizome of the medicinal plant turmeric<br>(*Curcuma longa*)	In vitro	SiHa, CaSki, HeLa, C33A	-	↓ HPV-18 transcription by selectively AP-1, which reverses the expression dynamics of c-fos and fra-1, Telomerase activity, Ras, ERK signaling pathways, Cyclin D1, COX-2, iNOS activity, Mitochondrial pathway, HPV E6 and E7 oncoproteins, NF-kB, Bcl-2, Bcl-XL<br>↑ Apoptosis through caspase-3; p21, ROS, Bax, AIF, release of cytochrome c, p53 (when in association with ellagic acid)<br>↑ sensitivity to vinblastine, mitoxantrone, and etoposide	[95–101,103,104]
		In vivo	phase I clinical trial	-	Histological improvements in 1 out of 4 cases.	
Cyanidin (Polyphenols, Flavonoids, Anthocyanidins)	Berries,<br>red fruits, black rice, some cereals, root vegetables	In vitro	HeLa	85.95-408.13 μg/mL	↓ Proliferation<br>↑ Dose- and time-dependent apoptosis through Bax/Bcl-2, ROS, Peroxides	[85,205]
Daidzein (Polyphenols, Flavonoids, Flavones)	Soy	In vitro	HeLa	-	↑ Human telomerase catalytic subunit mRNA decreased, Cell growth arrest, Cell cycle arrest, Telomerase activity	[78]
Decursin and Decursinol (Polyphenols, Pyranocoumarin compounds)	Roots of *Angelica gigas*	In vitro	Hela	5-10 μg/mL	↓ HPV E6 and E7 expression<br>↑ Apoptosis (TRAIL expression)	[135]
EGCG (Polyphenols, Catechins)	Green tea	In vivo	Clinical trial	-	Controls and promotes IL-23-dependent DNA repair<br>Modulates growth factor-mediated pathway, the mitogen-activated protein kinase-dependent pathway, and ubiquitin/proteasome degradation pathways<br>↓ Carcinogenic signal transduction pathways<br>↑ Cytotoxic T-cell activities	[64–70]
		In vitro and in vivo	CaSki	-	↓ Proliferation<br>↑ Dose-dependent apoptosis<br>Arrest of cell cycle in the G1 phase.	
		In vitro	CaSki, HeLa, C33A, TCL-1 (HPV-immortalized cervical epithelial cells), Me180	-	↑ Apoptosis, p53, Bcl-2 and p21 (dose-dependent)<br>↓ Proliferation (time- and dose- dependent, via regulating the expression of miRNAs), HPV E6/E7 expression, estrogen receptor α, aromatase by a time-dependent manner mediated by	

Compound	Dietary Sources	Model		IC50	Mechanism of Action	References
					apoptosis, Hypoxia- and serum-induced HIF-1α and VEGF (via MAPK and PI3K/AKT pathways)	
Ellagic acid Polyphenols, Hydroxybenzoic acids)	Longan (*Dimocarpus longan*), litchi (*Litchi chinensis*), walnuts, pecans, cranberries, raspberries, strawberries, grapes, and peaches	In vitro	HeLa, SiHa, C33A	< 1 µM; 48.7 µg/ml	↓ Cell viability, HPV E6 oncoprotein, STAT3 signaling, Cyclin D1, Bcl-x1, Mcl-1, Cell migration, Invasion, AKT/mTOR<br>↑ Apoptosis, Cell cycle arrest at G1/S/G2, p53, Bax, Caspase3, Caspase9, Cell growth arrest, enhanced expression of IGFBP7	[37]
		In vivo	Mice	-		
Eupatorin (Polyhenols, Flavonoids, Flavones)	Orthosiphon stamineus, Lantana montevidensis, and Tanacetum vulgare	In vitro	HeLa, CaSki	100-150 µM	↓ Cleaved caspase-3 expression, Proliferation, Hedgehog signal pathway, Angiogenesis<br>↑ Cell cycle arrest at G2/M, Cyclin B1, Cyclin D1, Ki67, p53, p21, Bax, Caspase-mediated apoptosis	[206–209]
Ferulic Acid (Polyphenols, Hydroxycinnamic acids)	Cereal grains, particularly the outer parts of the grain	In vitro	HeLa, ME-180	10 µg/ml	↓ Cell viability, MMP-9 mRNA expression, arrest in G0/G1 phase<br>↑ Efficacy of radiotherapy probably through ROS	[38,39]
Fisetin (Polyphenols, Flavonoids, Flavonols)	Cucumber, onion, persimmon, strawberry, apple	In vitro	HeLa	-	↓ Proliferation, Tumor growth, inhibition of ERK1/2<br>↑ Apoptosis (due to activation of the phosphorylation ERK1/2, inhibition of ERK1/2 by PD98059), Caspase-8-/caspase-3-dependent pathway	[72]
		In vivo	Immunodeficient nude mice	-		
Galangin (Polyphenols, Flavonoids, Flavonols)	*Alpinia officinarum*	In vitro	HeLa	12.5 µM	↓ Proliferation, Cell migration, Glyoxalase-1, Nfr-2<br>↑ ROS, Cell death	[210,211]
Gallic acid (Polyphenols, Hydroxybenzoic acids)	Blackberry, raspberry, walnuts, chocolate, wine, green tea, and vinegar	In vitro	HeLa, HTB-35, HUVEC	10-40 µM	↓ Cell viability, Proliferation, Invasion, Angiogenesis, Cytotoxicity on normal cells<br>↑ ROS and GSH depletion	[40,41]
Genistein (Polyphenols, Isoflavonids)	Soy, beans, chickpeas, alfalfa, and peanuts	In vitro	HeLa, CaSki	100 µM	↓ Cell migration by modulating MMP-9 and TIMP-1<br>↑ Cell growth arrest, Apoptosis, Cell cycle arrest at G2/M, Activity of cisplatin (a chemotherapeutic agent)	[85–89]
		In vivo	Agouti mice Sprague-Dawley rats	-		
Hesperetin (Polyphenols, Flavonoids, Flavanones)	Citrus fruit	In vitro	SiHa	650 µM	↓ Cell viability, Bcl-2<br>↑ Extrinsic and intrinsic apoptosis, Cytochrome c, Cleaved caspase-3, Cleaved caspase-8, Cleaved caspase-9, p53, Bax, FADD, Fas, Cell cycle arrest at G2/M	[42,43]
	Citrus fruit	In vitro	HeLa, SiHa, CaSki, C33A	-	↓ HPV E6 oncoproteins<br>↑p53, Cell cycle arrest at G2/M, Cell growth arrest	[43]

Compound	Dietary Sources	Model		IC50	Mechanism of Action	References
Hesperidin (Hesperetin-7-O-rutinoside)		In vivo	Xenograft mice, rats	-	↓ DNMT, HDAC, Cell proliferation<br>↑ Apoptosis, ER stress, Glucose uptake, ASK1/JNK pathway, ROS	
Isoliquiritigenin (Polyphenols, Isoflavonoids)	*Glycyrrhiza inflata* and Glycyrrhiza radix	In vitro	HeLa<br>CaSki, SiHa, C33A	39.9 – 53.76 – 58.10 – 32.83 μM	↓ Bcl-2, HPV E6 oncoprotein, p-p53, cdc25C, cdc2, Cyclin A, Cyclin B<br>↑ Apoptosis, Cell cycle arrest at G2/M, Caspase-3, Caspase-8, Cleavage of caspase-9, Caspase-12, PARP, Bax, p53, p21, Cytochrome c, p-eIF2α, GRP78 level, p-Chk2	[212,213]
Isorhamnetin (Polyphenols, Flavonoids, Flavonols)	*Ginkgo biloba, Persicaria thunbergii, Oenanthe javanica*, and *Hippophae rhamnoides*	In vitro	HeLa	100 μM	↓ Proliferation, Telomerase activity, Cdc25C, Cdc2, Cyclin B1<br>↑ Bcl-2, AMT-Chk2 pathway,<br>Cell cycle arrest at G2/M, Bax, Apoptosis, p-Cdc25C, p-Cdc2	[214–217]
Jaceosidin (Polyphenols, Flavonoids, Flavones)	Compositae	In vitro	SiHa<br>CaSki	-	↓ E6 and E7 functions (impairment of binding to p53 and pRb)<br>↑ caspase-3, caspase-9	[59,60]
Juglone (Polyphenols, Naphtoquinones)	*Juglans mandshurica*	In vitro	HeLa, CaSki, C33A	-	↓ Cell viability, Proliferation, Cell migration, Invasion<br>↑ Cell cycle arrest at G2/M, Morphological changes, Apoptosis, Cytochrome c, Caspase-3, PARP, p-JNK, p-c-Jun	[218,219]
Kaempferol (Polyphenols, Flavonoids, Flavonols)	Onions, oranges, and parsley	In vitro	HeLa, SiHa, CaSki, C33A, HaCaT	-	↓ Proliferation, Cyclin B1, CDK1, NF-κB nuclear translocation, Bcl-2, Bax<br>↑ Cell cycle arrest at G2/M	[73,74]
Luteolin (Polyphenols, Flavonoids, Flavones)	Celery, broccoli, green pepper, parsley, thyme, perilla, chamomile tea, carrots, olive oil, peppermint, rosemary, oranges and oregano	In vitro	CaSki, E6/E7 immortalized human foreskin keratinocytes (HFK), primary HFKs	23 μM	↓ E6 oncoprotein function (through binding mimicking leucines in the α-helical motif of E6AP)	[61]
		In vivo	HeLa	-	↑ Apoptosis (TRAIL-induced)	
Methylenedioxy lignan (Poyphenols, Lignans)	*Phyllanthus urinaria*	In vitro	HeLa	4.46 μM	↓ Cell proliferation, telomerase activity<br>↑ Apoptosis	[88]
Morin (Polyphenols, Flavonoids, Flavonols)	White mulberry, osage orange, apple guava, old fustic, strawberry, almond hull, figs, sweet chestnut, onions, jack fruit, red wine	In vitro	HeLa	214.28 μM	↓ Binding of HPV E6 oncoprotein to FADD and caspase-8, Proliferation, CDK1, Cdc25C, Survivin, Cyclin B1, CHK2, Bcl-2, Bcl-xL, AMPK, cIAP-1, cIAP-2, PKCε, and NF-kβ mRNA expression	[220,221]

Compound	Dietary Sources	Model		IC50	Mechanism of Action	References
					↑ Morphological changes, Cell cycle arrest at G2/M, p53, p21, Wee 1, Apoptosis, Death receptors pathway related genes mRNA expression, Bax, Bad, cytochrome c, Apaf-1, and caspases-9 genes mRNA expression, PI3K, AKT, mTOR, P70S6K, and Smac genes mRNA expression, ROS	
Myricetin (Polyphenols, Flavonoids, Flavonols)	Cranberry, dock, sweet potato leaves, chard, swiss, broad beans (immature seeds), rutabagas, garlic, blueberry, peppers (hot chili, green) blackberry, lotus root, and lemon	In vitro	HeLa	100 µM	LDH release<br>↓ Mitochondrial membrane potentials, Caspase-3<br>↑ Apoptosis, Cell cycle arrest at G0/G1	[222–224]
Naringenin and naringenin-loaded nanoparticles (Polyphenols, Flavonoids, Flavanones)	Grapefruit and citrus fruits	In vitro	SiHa, HeLa	750 µM	↓ Proliferation, Caspase-1, NF-κB p65 subunit, COX-2<br>↑ Apoptosis through both death-receptor and mitochondrial pathways, Cell cycle arrest at G2/M, Caspase-3, Caspase-9, p53, Bax, Fas, FADD<br>↑ Cytotoxicity (dose-dependent), ROS, lipid peroxidation<br>↓ Intracellular glutathione levels<br>Alterations in mitochondrial membrane potential	[44–49]
Nor-dihydro-guaiaretic acid (Polyphenols, Lignans)	*Larrea tridentata*	In vitro	SiHa	4.46 µM	↓ HPV E6 and E7 transcription and expression<br>↑ Apoptosis	[89]
Paeonol (Polyphenols, Hydroxybenzoketones)	*Cynanchum paniculatum*, and *Paeonia suffruticosa*	In vitro	HeLa	-	↓ Cell migration, Invasion, 5-lipoxygenase, Proliferation, PI3K/AKT signaling pathway<br>↑ Apoptosis, Morphological changes, ROS, Cytochrome c, Bax/Bcl-2 ratio, Caspase-3	[225,226]
Peonidin (Polyphenols, Flavonoids, Anthocyanidins)	Berries, red fruits, cereals, root vegetables	In vitro	HeLa	85.95 µM	↓ Proliferation<br>↑ ROS, Peroxides	[205]
Phloretin (Polyphenols, Chalcones)	Fruit, leaves, and roots of apple tree	In vitro	HeLa, CaSki, SiHa	-	↓ Cell viability, Cell migration, Invasion, Colony-forming capacity, Cathepsin S, MMP-2, MMP-3, Self-renewal ability, ALDH1 activity, Protease activities of cervical cancer-initiating cells<br>↓ Lung colonization, Tumor growth, Angiogenesis	[227]
		In vivo	SiHa xenograft mouse	-		
Pterostilbene (Polyphenols, Stilbenes)	Grapes, blueberries, red wine, peanuts, and some medicinal plants	In vitro	HeLa, PC1	15.61 µM	↓ HPV E6 Oncoprotein in vivo and in vitro, VEGF Protein in vivo<br>↑ Tumor development by cell cycle arrest and ↓ tumor growth<br>↑ Cleaved Caspase-3	[228,229]
		In vivo	HPV E6 Mice	-		

Compound	Dietary Sources	Model		IC50	Mechanism of Action	References
Puerarin (Polyphenols, Isoflavonoids)	*Pueraria lobata*, *Pueraria thomsonii* and *Pueraria tuberosa*	In vitro	HeLa	-	↓ Cell proliferation, P13K, p-Akt, p-mTOR<br>↑ Apoptosis	[230]
Punicalagin (Polyphenols, Hydroxybenzoic acids)	*Punica granatum*	In vitro	HeLa, ME-180	-	Modulating MMP-2, MMP-9, TIMP-2, and TIMP-3<br>↓ Cell viability, Bcl-2, Cell migration, β-catenin signaling pathway, Mitochondrial membrane potential<br>↑ ROS, Apoptosis, Bax, Caspase-3, Caspase-9, p53	[231]
Quercetin (Polyphenols, Flavonoids, Flavonols)	Onion, kale, leek, broccoli, buckwheat, red grapes, tea, and apples	In vitro	HeLa	110 µM	- Restores TSG expression fold change<br>↓ DNMTs, HDAC, HMT H3K9, 5′CpG promoter methylation of TSGs, Apoptosis, ROS, Cell cycle arrest at G2/M	[75–77]
Resveratrol (Polyphenols, Stilbenes)	Red wine, grapes, and berries	In vitro	SiHa, HeLa, C33A, CaSki	150-250 µM	↓ Proliferation, Invasion and migration by either inactivating phosphorylation of STAT3Tyr705 or ROS generation, HPV E6 oncoprotein, PCNA protein in vivo, VEGF protein in vivo<br>↑ Apoptosis, Autophagy, Fis1 and Deo1, ER stress, MiR-326/pyruvate kinase M2	[91–94]
		In vivo	Xenograft Mice	-		
Rutin (Polyphenols, Flavonoid glycosides)	Asparagus, buckwheat, apricots, apples, cherries, grapes, grapefruit, plums, oranges, and tea	In vitro	HeLa	30 µg/mL	↓ Proliferation, Tumor growth	[147]
		In vivo	K14-HPV-16 mice	-	↓ COX-2, Leukocytic infiltration in lesions	
Salvianolic Acid B (Polyphenols)	*Salvia miltiorrhiza*	In vitro	HeLa	-	↓ Cell viability, TNF levels<br>↑ Apoptosis	[232,233]
Scopoletin (Polyphenols, Hydroxycoumarins)	*Scopolia spp.*	In vitro	HeLa, SiHa, C33A, DoTc2	90 µM	↓ PI3K/AKT signaling pathway, Cell migration<br>↑ Cell growth arrest, Caspase-3, Caspase-8, Caspase-9, Apoptosis, DNA damages, Cell cycle arrest at G2/M	[234]
Sesamin (Polyphenols, Lignans)	Sesame oil	In vitro	HeLa	-	↓ Cell proliferation/migration<br>↑ Apoptosis (ER stress-mediated, through IRE1alpha/JNK)<br>Autophagy	[90]
Silibinin (Polyphenols, Flavonoids, Flavolignans)	milk thistle (*Silybum marianum*)	In vivo	HeLa	-	G2 arrest<br>↑ Apoptosis (dose- and time-dependent)	[86,87]
Wogonin	*Scutellaria baicalensis*	In vitro	SiHa, CaSki	-	↑ Apoptosis, p53 and pRb<br>↓ E6 and E7	[62,63]

Compound	Dietary Sources	Model		IC50	Mechanism of Action	References
(Polyphenols, Flavonoids, Flavones)						
Xanthohumol (Polyphenols, Flavonoids)	*Humulus lupulus*	In vitro	CaSki<br>HeLa	9.4 μM	↓ Bcl-2, XIAP, Cell proliferation, Mitochondrial membrane potential<br>↑ Cell cycle arrest at S, Caspase-3, Caspase-8, Caspase-9, PARP, p53, AIF, Apoptosis by both extrinsic and intrinsic apoptotic pathways, DNA fragmentations, Morphological changes, TRAIL-R2 protein levels	[235,236]
Alpha-carotene, beta-carotene, lutein, zeaxanthin and alpha-tocopherol (Terpenoids Carotenoids)	Carrot, kale, pumpkin, sweet potato	In vivo	Clinical trial on single-compounds or combination thereof	-	↓ risk of cervical cancer	[112]
Carnosic acid (Terpenoids, Diterpenoids)	Chinese medicinal herbs	In vitro	CaSki<br>SiHa	-	↓ Proliferation<br>↑ Cell growth arrest, Apoptosis, Caspase-3, Caspase-9, Cell cycle arrest at G2/M, ROS	[237]
		In vivo	CaSki xenograft, mouse	-	↓ Tumor growth	
Lycopene (Terpenoids, Carotenoids)	Tomato, watermelon, pink grapefruit, guava, papaia	In vitro	HeLa	-	↑ cells sensitivity to cisplatin<br>↓ viability<br>↑ Bax and Nrf2 expression<br>↓ Bcl-2 expression, NF-κB signaling path-way.	[113–115]
Saikasaponins (Terpenoids, Triterpens)	*Bupleurum falcatum*	In vitro	HeLa, SiHa	-	↑ sensitization to cisplatin (intracellular ROS accumulation)	[116,117]
Saponins (Terpenoids, Triterpens)	Chickpeas, soya beans, Chinese herbs	In vitro	HeLa	2.62 μM	↑ Apoptosis (cleaved Caspase-3 , Caspase-9, Bax/Bcl-2)	[118]
Tanshinone IIA (Terpenoids, Diterpenoids)	*Salvia spp*.	In vitro	HeLa, SiHa, CaSki, C33A	-	↓ Cell growth, HPV E6 and E7 expression<br>↑ Apoptosis, cell cycle arrest	[119]
Ursolic acid (Terpenoids. Triterpenoids)	Apples, cranberries, peppermint, prunes, oregano, thyme.	In vitro	CaSki, HeLa, C4-1, SiHa	-	↓ Cell proliferation, invation and migration (Caspases- and p53-mediated)<br>↓ Anti-apoptosis-related signals<br>↑ Apoptosis, expression of C/EBP homologous protein and glucose-regulated protein 78	[120,121]

Compound	Dietary Sources	Model		IC50	Mechanism of Action	References
		In vivo	CaSki, HeLa, SiHa xenografts	-	↓ Tumor size	
Allicin (Thiols - Allylic sulfides)	Garlic and onion	In vitro	SiHa	-	↑ apoptosis (inhibition of NRF2)	[122]
Indole-3-carbinol (I3C) and DIM, a stable form of I3C (Thiols – Indoles)	Broccoli and Brussels sprouts	In vitro	C33A	-	- Selective apoptosis induction	[123,129]
		In vivo	17-beta-estradiol (E2)-treated mice	-	↓ estradiol anti-apoptotic effect	
		In vivo	Randomized, double-blind, multi-center clinical trial	-	↓ CIN lesions (100% at 200 mg/day; 90.5% at 100 mg/day)	
Sulforaphane (Thiols, Glucosinolates)	Asparagus, broccoli, Brussel sprouts, cauliflower, horse-radish, mustard, radish	In vitro	HeLa, Cx and CxWJ	-	Cytotoxicity<br>G2/M phase arrest (dose-dependent, Cyclin B1 down-regulation)	[130,131]
Berberine (Alkaloids)	*Berberis genus*	In vitro	HeLa, SiHa	-	↓ Cell growth, HPV E6 and E7 expression, telomerase activity<br>↑ Apoptosis, cell cycle arrest, p53, pRB<br>Epigenetic modifications and disruption of microtubule network by p53 targeting	[132,133]
Betaines	Beetroot	In vitro	HeLa	-	↓ proliferation<br>G1/S or S/G2 cell cycle arrest<br>↑ Dose-dependent apoptosis (Bax, p53, caspase-3 and Bcl-2)	[134]
Piperine (1-pipeoylpeperdine)	*Piper nigrum*, *Piper longum*	In vitro	HeLa	-	↑ Dose-dependent apoptosis (caspase-3, loss of MMP, DNA fragmentation and ROS increase)	[136]
Withaferin A (Steroid lactone)	*Withania somnifera*	In vitro	CaSki	-	↓ HPV E6 and E7 expression<br>↑ Apoptosis	[137]
Lectins	*Astragalus mongholicus*	In vitro	HeLa	-	↓ Cell proliferation (↑ p21 and p27, ↓ active complex cyclin E/CDK2 kinase)<br>↑ Apoptosis	[139]
Peptides	*Triticum aestivum*, *Abrus precatorius*, *Trichosanthes kirilowii*	In vitro	HeLa	-	↓ Cell proliferation (↑ DNA damage, G2 arrest, ↓ CDK1-cyclin B1 complex, ↑ active chk1 kinase)<br>↑ Apoptosis (↑ ROS, ↓ Bcl-2/Bax ratio, ↑ mitochondrial permeability)	[140–142]

Compound	Dietary Sources	Model		IC50	Mechanism of Action	References
Polysaccharides	*Solanum nigrum*	In vitro and in vivo	U14	from 19 to 60 µg/ml	↓ Tumor growth (CD4+/CD8+ ratio modification)<br>↑ Apoptosis (TRAIL expression)	[238]
